# Palladium-Catalyzed Carbonylations: Application in Complex Natural Product Total Synthesis and Recent Developments

**DOI:** 10.1021/acs.joc.2c02746

**Published:** 2023-01-27

**Authors:** Hunter S. Sims, Mingji Dai

**Affiliations:** † Department of Chemistry, Emory University, Atlanta, Georgia30322, United States; ‡ Department of Chemistry, Purdue University, West Lafayette, Indiana47907, United States

## Abstract

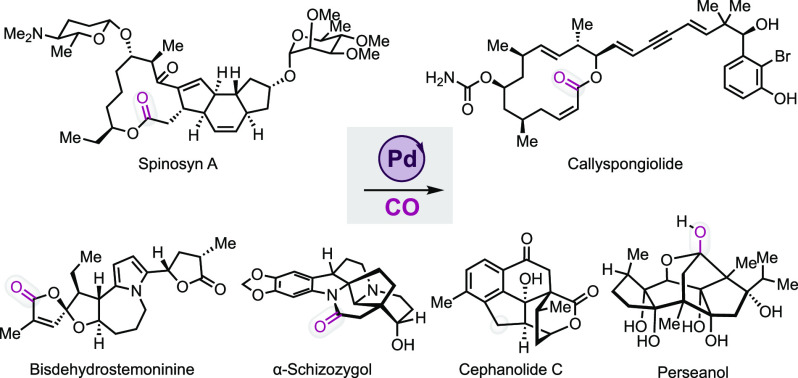

Carbon monoxide is a cheap and abundant C1 building block that can be readily incorporated into organic molecules to rapidly build structural complexity. In this Perspective, we outline several recent (since 2015) examples of palladium-catalyzed carbonylations in streamlining complex natural product total synthesis and highlight the strategic importance of these carbonylation reactions in the corresponding synthesis. The selected examples include spinosyn A, callyspongiolide, perseanol, schizozygane alkaloids, cephanolides, and bisdehydroneostemoninine and related stemona alkaloids. We also provide our perspective about the recent advancements and future developments of palladium-catalyzed carbonylations.

Among the C1 feedstocks, carbon monoxide is a cheap and abundant C1 building block of industrial importance. Various new reactions and technologies have been substantially developed in recent years to capture this important C1 feedstock.^1^ With the aid of transition-metal catalysis, carbon monoxide can readily be incorporated into organic molecules to introduce various functional groups and build structural complexity. In 1974, Heck and co-workers reported that activated halides can react with carbon monoxide and alcohols (or amines) to form esters (or amides) in the presence of a palladium catalyst.^2^ Since this seminal discovery, palladium-catalyzed carbonylation reactions have become a popular topic among the synthetic community with new and enabling methods constantly being developed.^3^ While other transition metals can also participate in carbonylation chemistry, palladium-based carbonylation methods have been the most diverse, extensively developed, and utilized in organic synthesis. For example, Heck-type carbonylation reactions are routinely used in medicinal chemistry and complex molecule synthesis to introduce esters and amides (or lactones and lactams through intramolecular trapping). Additionally, the Suzuki and Stille carbonylations are powerful methods for generating ketone products by uniting two building blocks with carbon monoxide as a one-carbon linchpin. When coupled with the Nazarov cyclization, cyclopentenone-containing products can be generated rapidly. The Semmelhack reaction^4^ has further expanded the application of palladium-catalyzed carbonylations to the construction of monocyclic and bicyclic *O*-heterocycles. Accordingly, it is to no surprise that palladium-catalyzed carbonylation reactions have found broad applications in natural product synthesis.^5^ In this Perspective, we first outline six recent (since 2015) complex natural product total syntheses that utilize palladium-catalyzed carbonylation reactions as key synthetic steps and highlight the strategic importance of each carbonylation in the corresponding synthesis. The selected examples include the following carbonylative processes: carbonylative macrolactonizations to spinosyn A^6^ and callyspongiolide,^7^ intramolecular Heck carbonylative lactonization to perseanol,^8^ intramolecular Heck carbonylative lactamization to schizozygane alkaloids,^9^ tandem carbonylative cyclization to cephanolides B and C,^10^ and oxaspirolactonization to bisdehydroneostemoninine and related stemona alkaloids.^11^ Following these total synthesis discussions, we offer our perspective about the recent developments of palladium-catalyzed carbonylations by highlighting key advancements made in the directions of sustainable carbonylations, photocatalytic carbonylation, C–H carbonylation, flow carbonylation, enantioselective carbonylation, carbonylative C11 incorporation, and new carbonylation reactions to build structural complexity.

## Palladium-Catalyzed Carbonylations in Streamlining Complex Natural Product Total Synthesis

### Spinosyn

**Figure 1:**
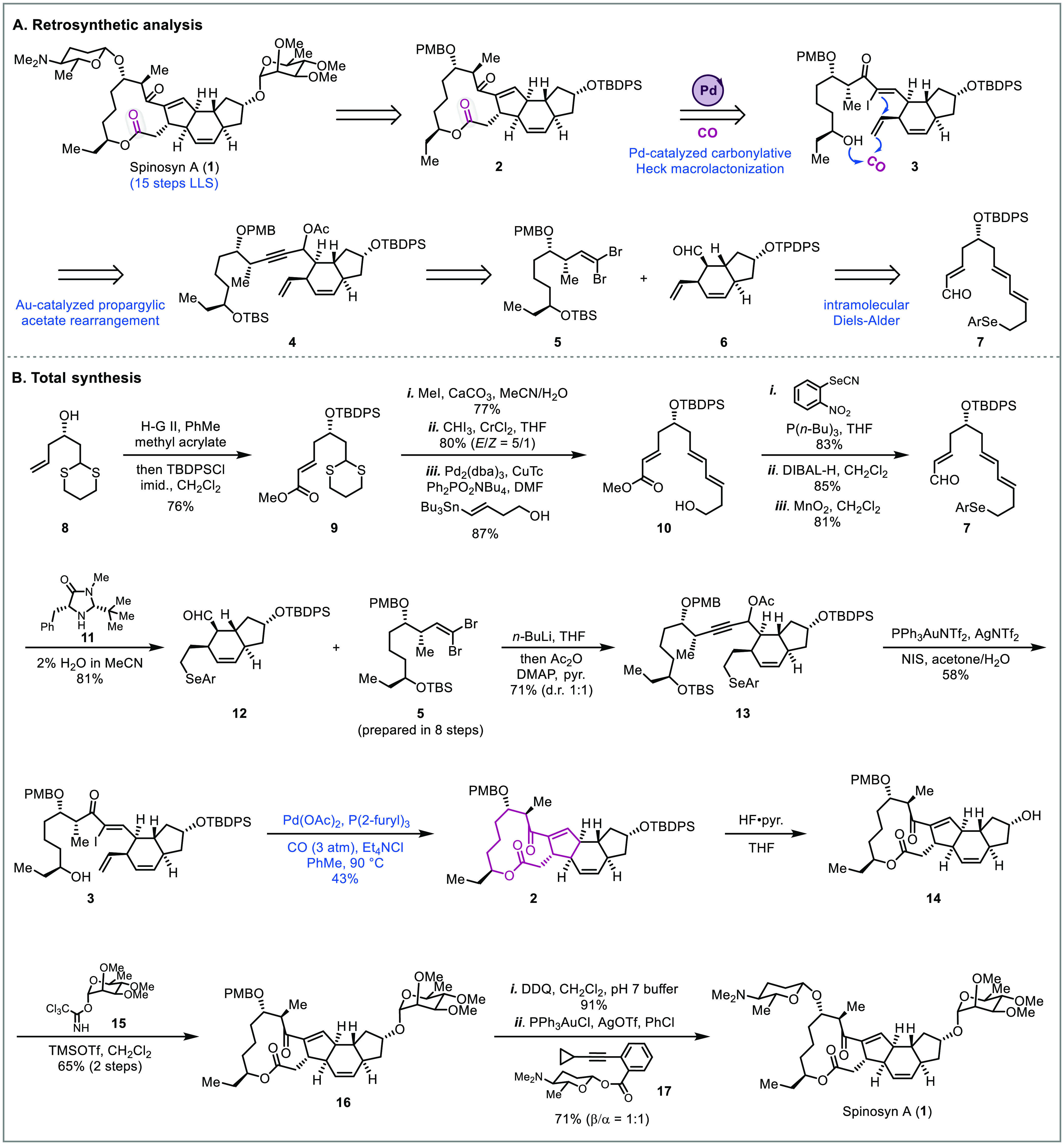
Total synthesis of spinosyn A (Dai, 2016).^6^

Spinosyns A and D, produced by *Saccharopolyspora spinosa* in a 17:3 ratio, are the major components of Spinosad which has been an important insecticide in agriculture.^12^ Additionally, it is FDA approved for the treatment of head lice owing to its very low mammalian toxicity. Spinosad primarily modulates the insect nicotinic acetylcholine receptor and also acts as a GABA neurotransmitter agonist. This combined effect results in nervous system hyperexcitation and eventual insect death. The identification of cross resistance among insects to Spinosad has prompted efforts toward the development of new derivatives with a broader insecticidal spectrum. Additionally, the distinct architecture and structural complexity of spinosyn A (**1**) has drawn the attention of many synthetic chemists,^13^ with elegant syntheses reported by the Evans,^14^ Paquette,^15^ Roush,^16^ and Liu^17^ groups. More recently, the Dai group developed a convergent synthetic route to (−)-spinosyn A (**1**) in only a 15 step longest linear sequence (LLS, Figure 1).^6^ To install the 12-membered macrolactone, a palladium-catalyzed carbonylative Heck macrolactonization of α-iodoenone **3** was proposed (Figure 1A), which would forge both the 5-membered carbocycle and the 12-membered macrolactone in a single step. A gold-catalyzed propargylic acetate rearrangement (**4** to **3**), a convergent coupling of fragments **5** and **6**, and a stereoselective intramolecular Diels–Alder reaction (**7** to **6**) would help to quickly build scaffold **3** for the key carbonylation step.

The Dai synthesis commenced with thio-ketal **8** (Figure 1B), which was converted to alcohol **10** through a cross-metathesis, TBDPS protection, thio-ketal deprotection, Takai olefination, and a Stille cross coupling. This intermediate was then transformed to **7** through selenide formation, DIBAL-H reduction, and MnO_2_ oxidation, which set the stage for a key chiral amine-catalyzed intramolecular Diels–Alder reaction (IMDA) based on the conditions reported by MacMillan and colleagues.^18^ The IMDA proceeded smoothly in the presence of amine **11** giving cyclized product **12** in 81% yield as a single diastereomer. This species was then coupled with fragment **5** (prepared in 8 steps) using the Corey–Fuchs protocol followed by *in situ* trapping of the resulting alkoxide with acetic anhydride to give **13**. Next, to install the α-iodoenone for the carbonylation cascade, the gold-catalyzed propargylic acetate rearrangement was employed. After extensive condition screening, it was found that oxidative selenide elimination, TBS deprotection, and the gold-catalyzed rearrangement could all be achieved in one pot to provide **3** in 58% yield. With the α-iodoenone in hand, the carbonylative Heck macrolactonization was attempted. The transformation could be achieved using palladium acetate as the catalyst and tri-(2-furyl)phosphine as the ligand under 3 atm carbon monoxide. This process proceeds through an oxidative addition of Pd(0) onto the α-iodoenone, 5-*exo*-trig carbopalladation, and CO migratory insertion to generate an acylpalladium intermediate which is subsequently trapped by the remotely tethered alcohol to give macrolactone **2**. Notably, during this process the internal double bond of the six-membered ring did not intercept the acylpalladium species. It is likely that the six-membered ring helped promote the desired cascade through the Thorpe–Ingold effect. With macrolactone **2** in hand, the total synthesis of spinosyn A **(1)** was achieved through the removal of protecting groups and attachment of the carbohydrate moieties. Unlike the conventional macrolactonization strategies that utilize *seco*-acid precursors, the palladium-catalyzed carbonylative Heck macrolactonization helped install both the 5-membered carbocycle and the 12-membered macrolactone in just one step. Since the highly reactive acylpalladium species was generated *in situ* and trapped with the tethered secondary alcohol, no carboxylate synthesis and activation were required. Thus, the palladium-catalyzed carbonylative macrolactonization significantly enhanced the overall synthetic efficiency.

### Callyspongiolide

**Figure 2:**
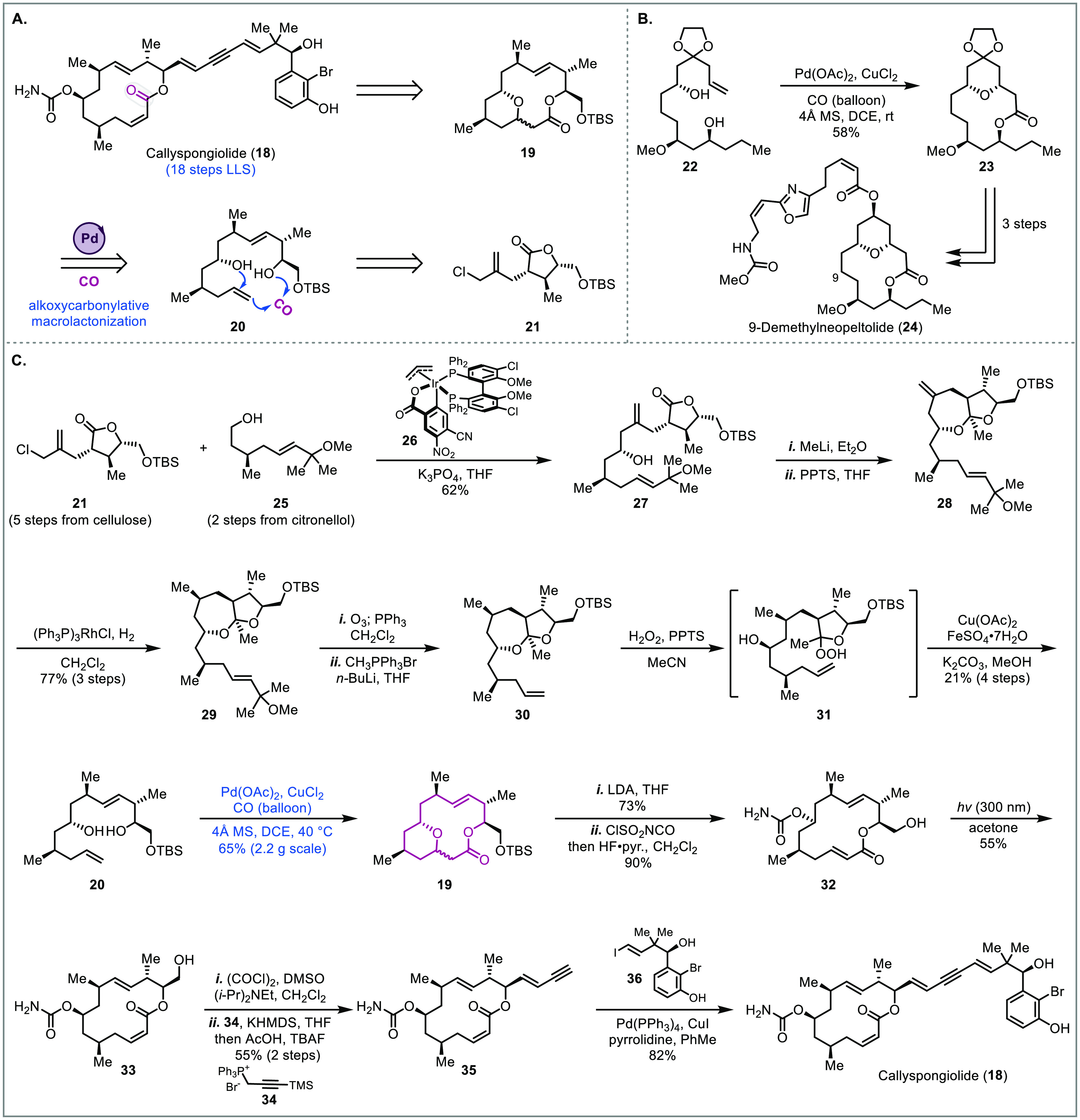
Total synthesis of callyspongiolide (Harran, 2018).^7^

Callyspongiolide (**18**), isolated from *Callyspongia* sp. in 2014, is a polyketide natural product that bears a distinctive phenethylated diene–ynic side chain.^19^ Callyspongiolide exhibits caspase-independent cytotoxicity in Jurkat and Ramos B lymphocyte cell lines, which indicates that it induces cell death via a nonapoptotic pathway. In 2018, the Harran group reported a creative 18-step total synthesis of (−)-callyspongiolide (Figure 2).^7^ Unlike previous approaches toward this natural product,^20^*seco*-acid precursors were not used to build the macrolide. Instead, the route relied on a palladium-catalyzed alkoxycarbonylative macrolactonization that installed a tetrahydropyran (THP) ring and a macrolactone in one step (**20** to **19**, Figure 2A). Mechanistically, this transformation proceeds through a 6-*exo*-trig oxypalladation of the olefin to give an alkyl-palladium bound THP intermediate, which then undergoes carbon monoxide migratory insertion and subsequent trapping with the tethered alcohol to form the macrolactone. This Semmelhack-type macrolactonization was first developed by Dai and co-workers and used in their total synthesis of 9-demethylneopeltolide to build the THP-bridged macrolactone (Figure 2B).^21^ Notably, the Semmelhack alkoxycarbonylation has been widely used in total synthesis.^5,22^ For example, in 2011 Yang and co-workers disclosed an elegant total synthesis of schindilactone A by using a modified Semmelhack alkoxycarbonylation protocol to install a key THP-fused γ-lactone system.^22a^ The conditions they developed were later used by both Wong^22b^ and Carreira^22c^ in their pallambin total synthesis. However, the application of the Semmelhack alkoxycarbonylation was previously limited to form esters or small-sized lactones. The two cases from Dai and Harran demonstrated its potential in building THP/THF-bridged macrolactones directly.

The Harran synthesis commenced with a diastereoselective iridium-catalyzed coupling of fragment **21** (synthesized from cellulose powder) and fragment **25** (synthesized from citronellol) to give **27** in 62% yield with excellent selectivity (dr >95:5) (Figure 2C). Next, methyl lithium addition to the lactone and subsequent ketalization gave *cis*-fused dioxaperhydroazulene **28**, which smoothly underwent regio- and stereoselective hydrogenation with Wilkinson’s catalyst to provide **29**. Subsequent ozonolysis, Wittig olefination, and perhemiketal formation with hydrogen peroxide then gave **31**, setting the stage for a unique perhemiketal fragmentation. In the presence of Cu(OAc)_2_ and FeSO_4_, fragmentation of **31** proceeded well followed by *in situ* deacylation to afford homoallylic alcohol **20** as a single olefin isomer. The resulting olefin geometry and regiochemical outcome for this process can be rationalized by the reaction mechanism. Generation of an alkoxy radical by iron followed by fragmentation of the α–β C–C bond generates a carbon-centered radical. Oxidative coupling of this radical with Cu(OAc)_2_ then produces an alkyl copper intermediate, which undergoes syn coplanar β-hydride elimination to the final product. With the macrolactonization precursor in hand, the palladium-catalyzed alkoxycarbonylation was employed. With only minor modifications to the previously reported conditions^21^**19** was delivered in 65% yield at gram scale as an inconsequential mixture of diastereomers. This mixture was then treated with LDA, which readily opened the tetrahydropyran ring via β-alkoxy elimination. After a one-pot carbamoylation of the secondary alcohol with ClSO_2_NCO and TBS deprotection of the primary alcohol, macrolactone **32** was isolated in good yield but as the *E*,*E* geometric isomer. Since the natural product contains the *Z*,*E* isomer, photoequilibration was used to convert the *E*-acrylate to the *Z*-isomer. Under photochemical conditions, the desired *Z* product **33** was isolated in 55% yield, while the recovered *E*-product could be resubjected to the same conditions. Callyspongiolide (**18**) was then obtained after Swern oxidation, Wittig olefination with **34** to the enyne, deprotection of the TMS-alkyne, and Sonogashira coupling with vinyl iodide **36**. Overall, this synthesis represents an unconventional approach to synthesize macrolide natural products. Even though there is no THP ring in the target molecule, the palladium-catalyzed alkoxycarbonylative macrolactonization coupled with the β-alkoxy elimination offered a creative and efficient approach to constructing the macrolactone of **18**.

### Perseanol

**Figure 3:**
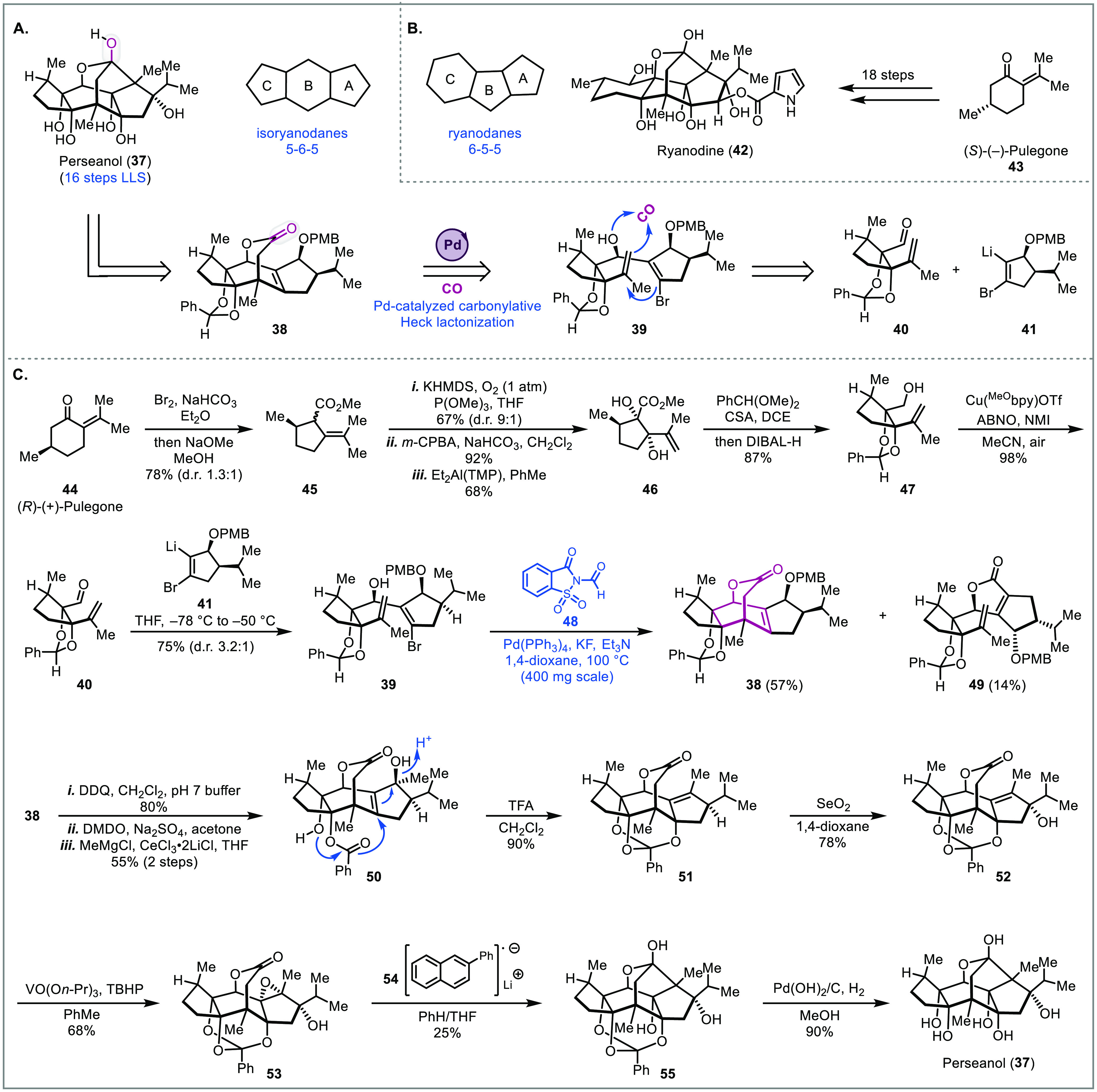
Total synthesis of perseanol (Reisman, 2019).^8^

The isoryanodane (represented by perseanol (**37**), Figure 3A) and ryanodane (represented by ryanodine (**42**), Figure 3B) diterpenes are complex natural products that possess insecticidal and antifeedant activities.^23^ While **42** has been shown to target ryanodine receptors (ligand-gated ion channels critical for intracellular Ca^2+^ signaling), perseanol was not confirmed to modulate this target indicating that it potentially has a different mode of action. This could in part explain why both **37** and **42** have potent activities but only perseanol exhibits low toxicity toward mammalian cells. In 2017, the Reisman group reported an elegant 18-step synthesis to ryanodine (Figure 3B).^24^ Following this work, they later reported a remarkable 16-step synthesis of (+)-perseanol in 2019 (Figure 3A).^8^ One of their key steps to quickly build the perseanol scaffold relied on an intramolecular carbopalladation-carbonylation cascade to close two rings (**39** to **38**). This efficient process proceeds through an oxidative addition of Pd(0) onto the alkenyl bromide, followed by a 6-*exo*-*trig* carbopalladation to give an σ-alkylpalladium species. This intermediate, which is not capable of β-hydride elimination, further undergoes carbon monoxide migratory insertion to an acylpalladium species. Subsequent trapping of the acylpalladium species with a proximal secondary alcohol furnishes lactone **38**. To access this intermediate, a convergent approach was taken via coupling of fragments **40** and **41**.

Starting from pulegone **44**, the fully elaborated C-ring precursor fragment **40** was prepared in six steps (Figure 3C). An oxidative ring contraction of pulegone gave **45** as a mixture of inconsequential stereoisomers. α-Hydroxylation, epoxidation, and a diethylaluminum 2,2,6,6-tetramethylpiperidide (Et_2_Al(TMP)) promoted epoxide ring opening afforded diol **46**, which was further advanced to **47** via a one-pot diol protection and DIBAL-H reduction. Lastly, a copper-catalyzed aerobic oxidation completed the six-step sequence to prepare aldehyde **40**, which was next treated with alkenyllithium **41** generated through a selective lithium iodide exchange to give key intermediate **39** in good yield and moderate diastereoselectivity, setting the stage for the carbonylation cascade. Initial studies were conducted under a CO atmosphere, however, nearly full starting material recovery was observed. Control studies indicated that carbon monoxide was inhibiting the oxidative addition step. This was further supported by experiments in which good yields were obtained by stirring **39** with stoichiometric amounts of the palladium catalyst followed by introduction of the CO atmosphere. To address this issue, a CO surrogate was employed that would help maintain a low CO concentration. After comprehensive reaction condition screening, they found that *N*-formylsaccharin **48** as the surrogate in combination with potassium fluoride and triethylamine was optimal for the carbonylative transformation. Although 50 mol % of Pd(PPh_3_)_4_ was required, lactone **38** was obtained in satisfactory yield (57%) as a single diastereomer. Additionally, under these conditions only 14% of premature carbonylation product **49** was produced. Subsequent PMB deprotection with DDQ, oxidation of the allylic alcohol to an enone with DMDO, and 1,2-addition to the resulting enone with MeMgCl gave intermediate **50**. Interestingly, when excess DMDO was used, the acetal was also oxidized to the hydroxybenzoate. Fortuitously, it was discovered that under acidic conditions hydroxybenzoate **50** could undergo a 1,3 allylic transposition to a dioxolenium ion, which was subsequently trapped by the proximal secondary alcohol to give orthobenzoate **51**. This intermediate was then progressed to perseanol (**37**) through a sequence of allylic oxidation, hydroxy directed epoxidation, reductive cyclization using lithium 2-phenylnaphthalenide **54**, and hydrogenation of the orthobenzoate. Altogether, the palladium-catalyzed carbonylation step served as a key step to build the central [3.2.2] bicyclic structure in just one step.

### Schizozygane Alkaloids

**Figure 4:**
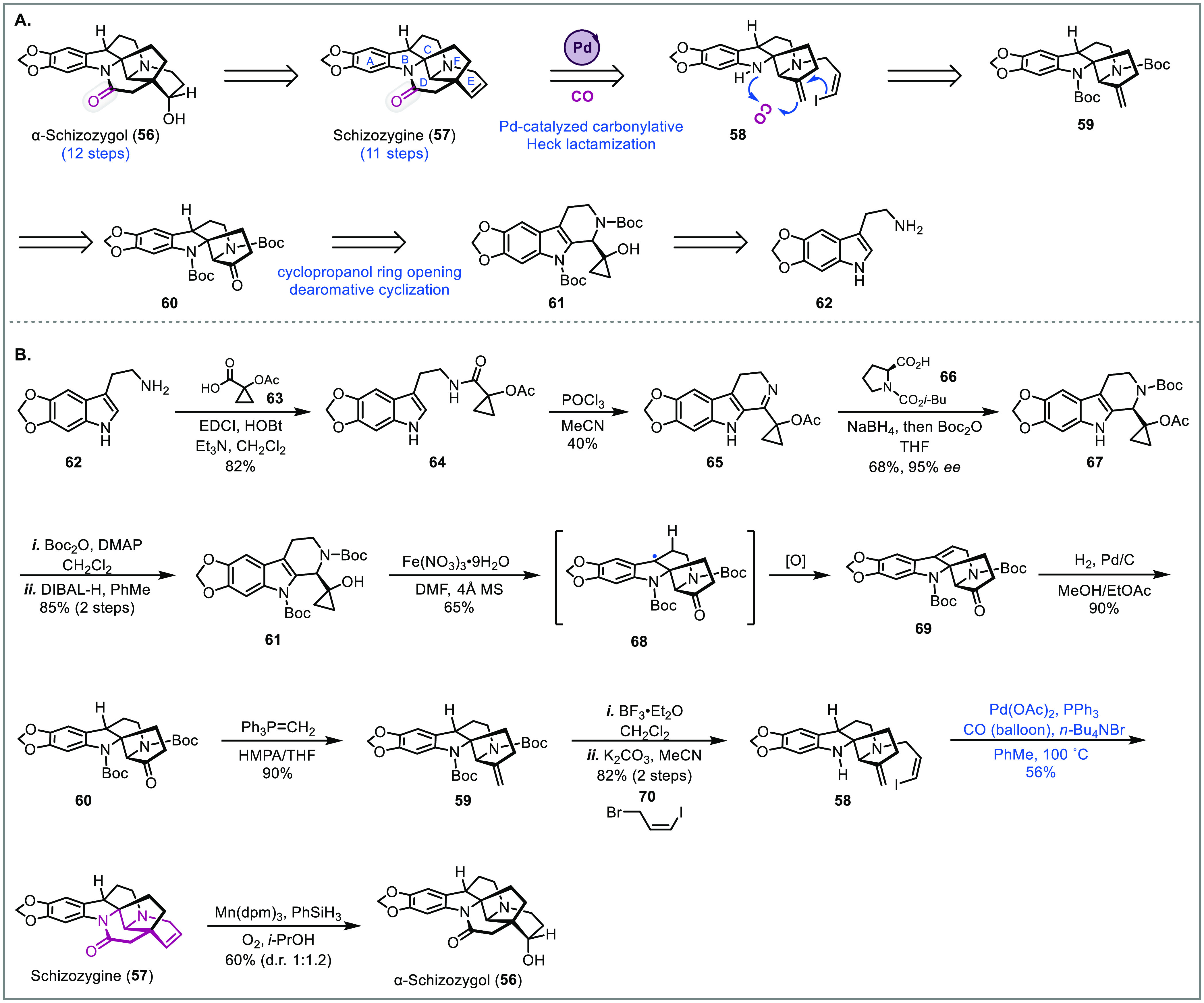
Total syntheses of schizozygane alkaloids (Zhang, 2021).^9^

The schizozygane alkaloids are rearranged monoterpene indole alkaloids. With the recent discovery that many of these natural products possess antiplasmodial and antifungal activities,^25^ there has been a renewed interest in the isolation, total synthesis, and biological testing of new schizozygane members. Accordingly, in 2021 the Zhang group reported asymmetric total syntheses of (+)-α-schizozygol (**56**), (+)-schizozygine (**57**), and several other schizozygane alkaloids (Figure 4A).^9^ These alkaloids contain up to six contiguous stereocenters, two of which are quaternary, and a sterically congested “Pan lid”-like scaffold with an embedded tertiary amide. Analogous to the Reisman carbonylative lactonization cascade toward perseanol, a Heck-carbonylative lactamization cascade was proposed by Zhang et al. which would construct two rings in one step, install the tertiary amide, and correctly place the endocyclic olefin of schizozygine (**57**). The olefin could be used as a handle to synthesize other schizozygane alkaloids such as α-schizozygol (**56**). Additionally, a creative approach to the ABCF ring system was planned through an oxidative dearomative cyclization of a tethered cyclopropanol onto the indole core.

The synthesis commenced with the preparation of imine **65** from tryptamine derivative **62** through an EDCI coupling with carboxylic acid **63** followed by a Bischler–Napieralski cyclization. Unfortunately, asymmetric transfer hydrogenation of **65** using the Noyori–Ikariya catalyst was unsuccessful. However, reduction using a proline **66** derived triacyloxyborohydride followed by *in situ* Boc protection of the secondary amine delivered **67** in good yield and enantioselectivity. Protection of the indole and removal of the cyclopropanol acetyl group then gave **61** for the dearomative cyclization. Using Fe(NO_3_)_3_ hydrate, the desired product **69** was obtained in 65% yield. The addition of molecular sieves was necessary to prevent trapping of the carbocation intermediate with water. Subsequent steps involving hydrogenation of the internal olefin, Wittig olefination of the ketone, Boc deprotection, and alkylation with bromide **70** gave precursor **58** for the Heck-carbonylative lactamization cascade. After extensive condition screening, they found that preheating the substrate with a stoichiometric amount of Pd(OAc)_2_ in toluene with PPh_3_ and *n-*Bu_4_NBr, followed by exposure to a CO atmosphere furnished schizozygine (**58**) in 56% yield. Schizozygine was then converted to α-schizozygol **(56)** through Mukaiyama hydration of the double bond (dr 1:1.2) albeit as the minor diastereomer.

### Cephanolides

**Figure 5:**
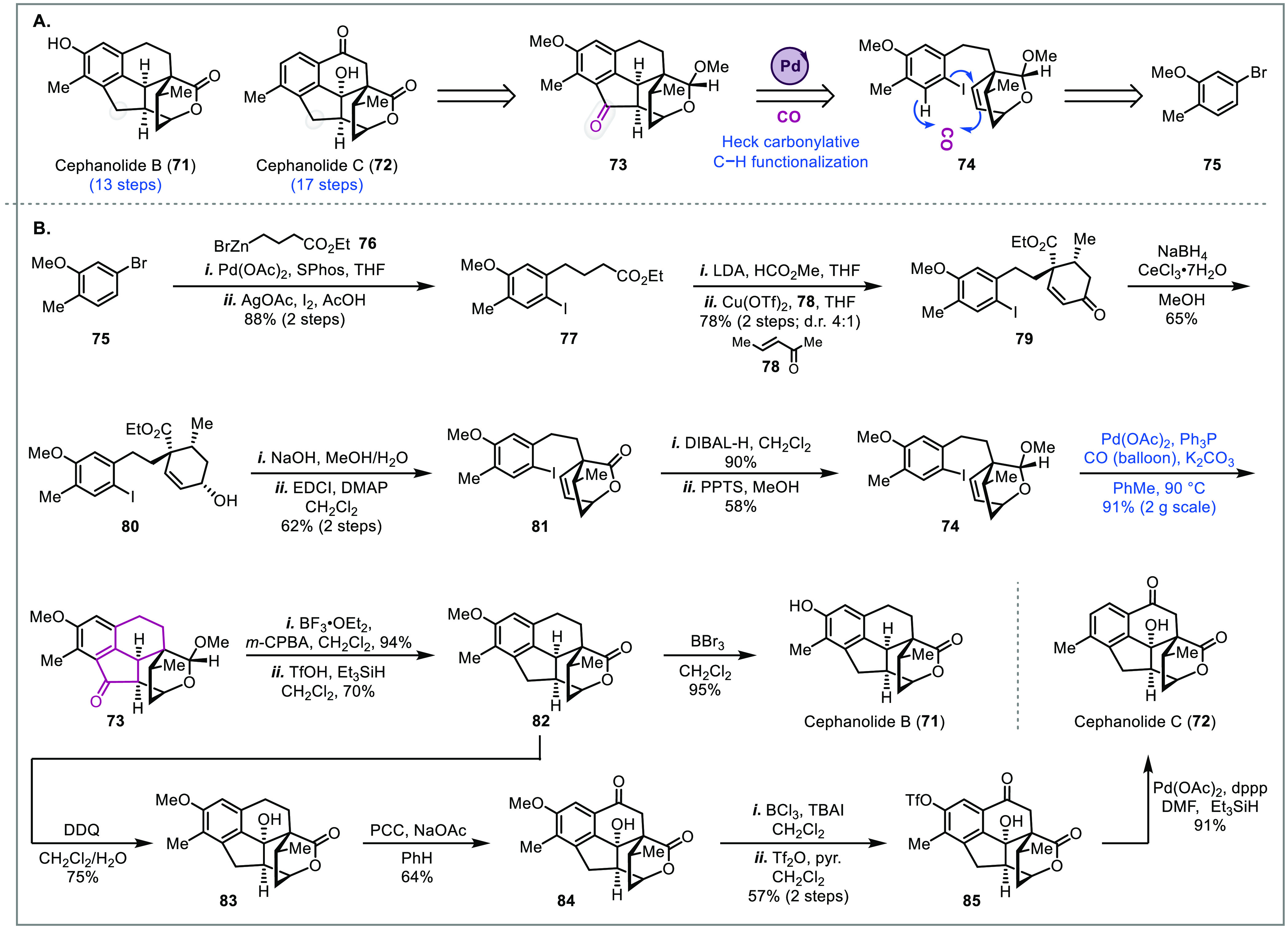
Total syntheses of cephanolides B and C (Zhao, 2018).^10^

The *Cephalotaxus* natural products have been attractive target molecules for total synthesis.^26^ In addition to their intriguing molecular architectures, many display potent anticancer activity. Notably, the alkaloid homoharringtonine was approved by the FDA for chronic myeloid leukemia.^27^ Thus, synthetic efforts toward newly isolated *Cephalotaxus* derivatives could facilitate the development of potent therapeutic candidates. In 2017, Yue and colleagues isolated four new structurally distinct norditerpenoids, cephanolides A–D, containing a benzenoid ring.^28^ Soon after, Zhao and co-workers reported efficient racemic syntheses of cephanolides B (**71**) and C (**72**) utilizing a Heck-type carbonylative C–H functionalization cascade to forge the benzo-fused ketone. Notably, this cyclization generates three C–C bonds and two new stereocenters (**74** to **73**, Figure 5A) in a single step.^10^ They went on to demonstrate that this transformation could be expanded to ether- and lactone-bridged bicyclic alkenes and to electron-deficient benzenoid rings albeit in lower yield. Mechanistically, this process proceeds through a Heck-type cyclization followed by carbon monoxide migratory insertion to an acylpalladium intermediate. While several possible mechanisms could terminate the catalytic cycle, on the basis that electron-rich benzenoids gave higher yields for the transformation, it was proposed that the final product was generated through Friedel–Crafts acylation of the acylpalladium intermediate.

Starting from commercially available bromide **75**, Negishi coupling with alkyl zinc bromide **76** and C–H iodination of the aryl ring gave intermediate **77** (Figure 5B). Next, α-formylation of the ester followed by a Robinson annulation with 3-penten-2-one **78** delivered enone **79** in good yield and moderate diastereoselectivity. After Luche reduction of ketone **79**, a sequence of saponification and lactonization gave rise to bridged bicyclic lactone **81**, which could then be transformed to key intermediate **74** through partial reduction to the aldehyde and subsequent acetalization. The Heck-type carbonylative C–H functionalization cascade then proceeded smoothly on a 2 g scale to deliver **73** as a single diastereomer. Utilization of acetal **74** was imperative for high stereoselectivity since the methoxy group can block the undesired face of the alkene. When **81** was used under the carbonylation conditions, the face of the alkene proximal to the lactone was less hindered and the opposite (and undesired) stereoselectivity was observed at the newly formed ring junction. Product **73** was then transformed to intermediate **82** through oxidation of the acetal to the lactone and ionic reduction of the ketone to a methylene. Intermediate **82** was used to access both cephanolides B (**71**) and C (**72**). Cephanolide B (**71**) was directly synthesized through demethylation of the benzenoid methoxy group with BBr_3_. On the other hand, five steps were required to transform **82** to cephanolide C (**72**). This sequence started with two benzylic C–H oxidations to give **84** followed by demethylation of the benzenoid methoxy group with BCl_3_. The resulting phenol underwent subsequent triflation to provide **85**, which underwent a palladium-catalyzed reduction of the triflate to **72**. The efficiency of Zhao and colleagues’ route to these natural products can be attributed to the remarkable Heck-type carbonylative C–H functionalization cascade that builds the bridged ring system in a single step on gram-scale.

### Stemona Alkaloids

**Figure 6:**
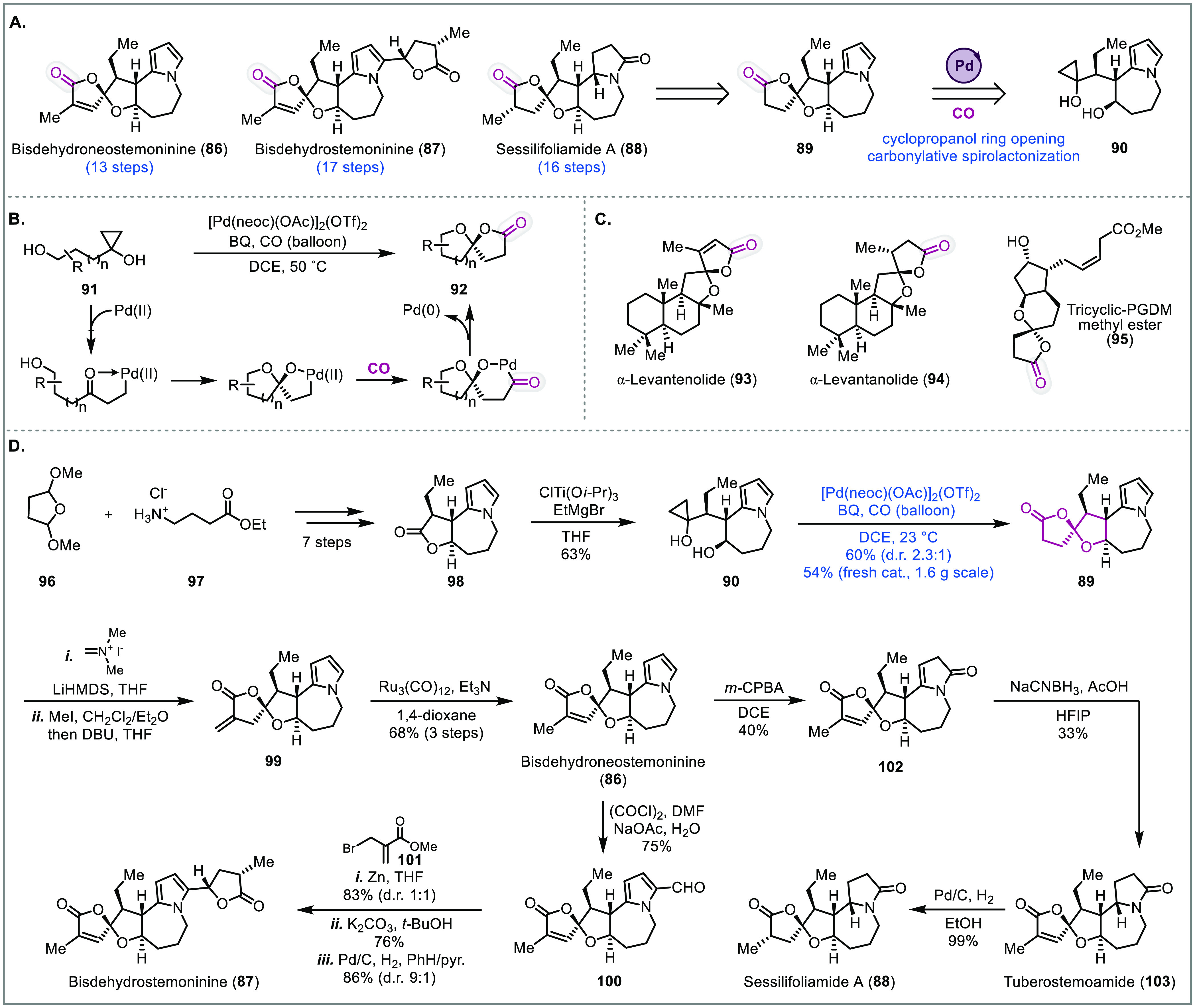
Total syntheses of stemona alkaloids (Dai, 2018, 2020).^6^

The Stemonaceae plants are an abundant source of bioactive natural products, and have been popular in Chinese and Japanese traditional medicines as a cough suppressant and insecticide. With over 150 stemona alkaloids isolated so far, they are further categorized into eight different groups with the majority containing a pyrrolo[1,2-*a*]azepine nucleus.^29^ In the stemoamide group, many members contain an oxaspirolactone moiety such as bisdehydroneostemoninine (**86**), bisdehydrostemoninine (**87**), or sessilifoliamide A (**88**) (Figure 6A).^30^ The Dai group has been interested in oxaspirolactone derived natural products. They have developed a palladium-catalyzed carbonylative oxaspirolactonization method to access such natural products (Figure 6B).^31^ The method converts hydroxycyclopropanols (cf. **91**), which can be easily accessed from the corresponding ester or lactone through the Kulinkovich reaction, directly to oxaspirolactones in a single step under mild conditions. For this carbonylative process, after coordination with the cyclopropanol, the Pd(II) catalyst promotes cleavage of the strained three membered cyclopropanol ring to generate a palladium-homoenolate. The tethered alcohol then cyclizes on the newly formed ketone to generate a ketal, which upon carbon monoxide insertion and lactonization of the resulting acyl-palladium species generates the oxaspirolactone product and Pd(0). The latter is then oxidized back to the Pd(II) catalyst with an external oxidant such as benzoquinone to restart the catalytic cycle. This transformation has been used by Dai and co-workers to synthesize several oxaspirolactone-containing natural products such as α-levantenolide (**93**), α-levantanolide (**94**),^31^ and tricyclic-PGDM methyl ester (**95**)^32^ (Figure 6C). They also used this transformation to build the oxaspirolactone moiety of the stemona alkaloids (**90** to **89**, Figure 1A) and completed the total synthesis of bisdehydroneostemoninine (**86**), bisdehydrostemoninine (**87**), sessilifoliamide A (**88**), and tuberostemoamide (**103**).^11^

Their synthesis commenced with commercially available starting materials **96** and **97**, which were converted to tricyclic lactone product **98** in seven steps (Figure 6D). To set the stage for the carbonylative oxaspirolactonization, conversion of **98** to hydroxycyclopropanol **90** was investigated. However, initial attempts were unfruitful. Under the standard Kulinkovich conditions, only meager yields were obtained presumably due to the steric hindrance around the lactone. In the Corey total synthesis of isoedunol, a hindered lactone was efficiently converted to a cyclopropanol using the more electrophilic and less hindered ClTi(O*i-*Pr)_3_ in place of Ti(O*i-*Pr)_4_.^33^ Under the Corey modified conditions cyclopropanol **90** was synthesized in good yield. Next, the key carbonylative oxaspirolactonization proceeded smoothly to give the desired diastereomer in moderate excess. While TFA could be used to epimerize the undesired diastereomer, it was later found that using the freshly prepared Waymouth palladium catalyst could give **89** in 54% yield as a single isomer on gram scale. Oxaspirolactone **89** was then carried to bisdehydroneostemoninine (**86**) using the Eschenmoser protocol to install an α-*exo*-methylene followed by isomerization to the endocyclic double bond using catalytic Ru_3_(CO)_12_. Bisdehydroneostemoninine (**86**) could then be transformed to bisdehydrostemoninine (**87**) in four steps. In subsequent work, Dai and co-workers also converted bisdehydroneostemoninine (**86**) to tuberostemoamide (**103**) and sessilifoliamide A (**88**) by transforming the pyrrole group to a lactam via a *m*-CPBA oxidation followed by reduction. In these total syntheses, the palladium-catalyzed carbonylative oxaspirolactonization provided an efficient and reliable way to build the oxaspirolactone moiety of the target molecules.

### Perspective of Recent Developments

Herein, six recent total syntheses were discussed that highlight the importance of palladium-catalyzed carbonylation reactions in facilitating the design and execution of complex natural product total synthesis. Apparently, the impact of palladium-catalyzed carbonylation chemistry is not limited to natural product total synthesis. Their applications expand into numerous other areas such as the chemical industry, pharmaceutical industry, agrosciences, material chemistry, and others. Meanwhile, there are still many limitations of the palladium-catalyzed carbonylation chemistry. Further improvements are strongly needed and are currently under investigation by many research groups. In the following part, recent advancements made in sustainable carbonylation, photocatalytic carbonylation, C–H carbonylation, flow carbonylation, enantioselective carbonylation, carbonylative C11 incorporation, and new carbonylation reactions to build structural complexity are highlighted.

### Sustainable Carbonylation

**Figure 7:**
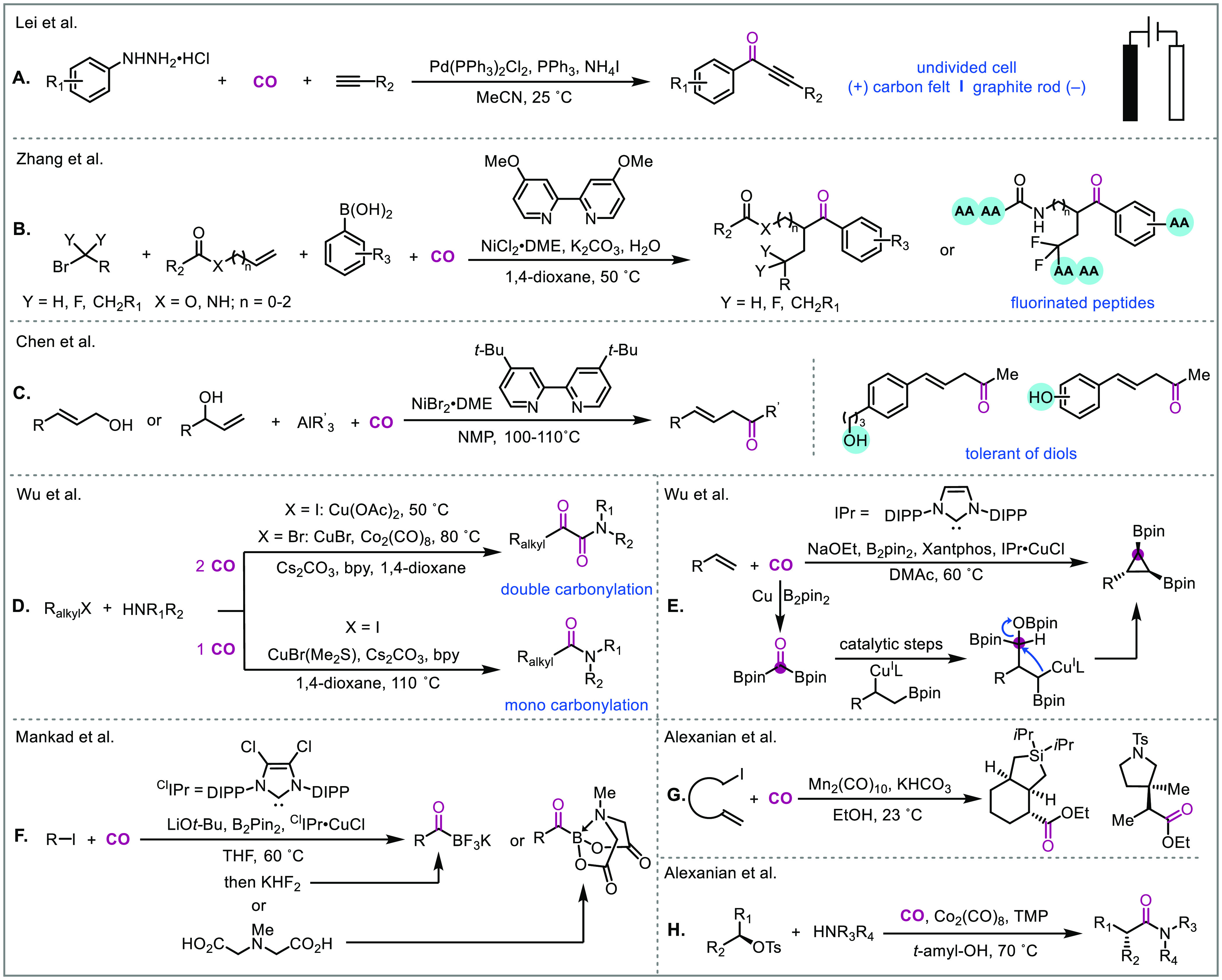
Recent examples of first-row transition metal carbonylative transformations.

Palladium is unparalleled in its ability to facilitate a diverse range of transformations in a predictable manner. However, the cost of palladium and the routinely needed high catalyst loadings are obvious drawbacks that limit the application of palladium-catalyzed carbonylation. One major reason for the requirement of high palladium catalyst loadings is the fast formation of palladium black (catalyst bleach) under carbon monoxide. Thus, the development of new ligands and more stable catalyst systems are important to solve the high catalyst loading issue.^3^ Also, finding more benign oxidant systems is highly sought after for oxidative carbonylations. In these reactions, the resulting Pd(0) must be oxidized back to Pd(II), which is usually achieved with an external oxidant such as a benzoquinone or a copper salt.^34^ Numerous examples have been reported that use oxygen (or air) as an oxidant or co-oxidant in conjunction with a copper salt, but for industrial applications this can raise the risk of an explosion. Electrochemical oxidation is an environmentally friendly solution which operates under mild conditions without the need for an external oxidant.^35^ Accordingly, electrochemical carbonylation methods are already starting to be developed. For example, in 2021 the Lei group developed an electrochemical palladium-catalyzed oxidative carbonylation of arylhydrazines and alkynes to ynones (Figure 7A).^36^ Their method operates under mild conditions (1 atm CO) and was used to build numerous ynone products from biologically active scaffolds. In addition to developing more sustainable palladium systems, using first-row transition metals such as nickel,^37^ copper,^38^ iron,^39^ manganese,^40^ and cobalt^41^ is particularly attractive since they are more abundant and significantly cheaper than palladium. However, this metal swap tactic is nontrivial and requires strategic experimental design and extensive catalyst screening. Nonetheless, progress is being made in this direction. For example, there has been a recent surge in nickel-catalyzed carbonylations without using the notoriously toxic Ni(CO)_4_. Notably, the Zhang group recently designed a nickel-catalyzed four-component carbonylation protocol for alkenes which could be further applied to the synthesis of fluorinated amino acids and oligopeptides (Figure 7B).^37a^ Soon after, the Chen group developed a nickel-catalyzed three-component carbonylative cross-coupling of allylic alcohols with organoalanes and CO to deliver high value β,γ-unsaturated ketones (Figure 7C).^37b^ Interestingly, the organoalanes could act as both a coupling partner and an activator for the allylic alcohol substrates preventing the need for extrinsic activators. Additionally, copper-, manganese-, and cobalt-catalyzed carbonylations are being developed. For example, Wu and colleagues very recently reported a copper-catalyzed highly selective double carbonylation of alkyl bromides to α-keto amides (Figure 7D).^38c^ For alkyl iodides, mono or double carbonylation could be controlled by modifying the reaction conditions. High value borylated building blocks can also be built through carbonylation. In line with this, Mankad and colleagues developed a copper-catalyzed carbonylative borylation of alkyl halides to give acyl boron intermediates, that could be further transformed to acyltrifluoroborates (KATs) and *N*-methyliminodiacetyl (MIDA) acrylboronates in one pot (Figure 7F).^38e^ In addition to being a powerful tool for the preparation of carbonyl scaffolds, transition-metal-catalyzed carbonylation can also be used as a one-carbon linchpin to build other complex scaffolds. For example, in 2020 Wu, Marder, and colleagues discovered a remarkable copper-catalyzed one-pot synthesis of cyclopropyl bis(boronates) from alkenes using CO as the C1 source (Figure 7E).^38d^ In the reaction, carbon monoxide insertion forms a bis(boryl)ketone, which does not act as a carbonyl precursor. Instead, through several copper-catalyzed steps the bis(boryl)ketone reacts with an alkyl copper intermediate (derived from an alkene) to give the cyclopropyl bis(boronate) product and a copper bound borate which can re-enter the catalytic cycle. Manganese- and cobalt-catalyzed carbonylations are starting to become more prevalent as well. For example, the Alexanian group reported a manganese-catalyzed carboacylation of alkenes (Figure 7G)^40a^ and a cobalt-catalyzed stereospecific aminocarbonylation of alkyl tosylates (Figure 7H).^41a^

### Photocatalytic Carbonylation

**Figure 8:**
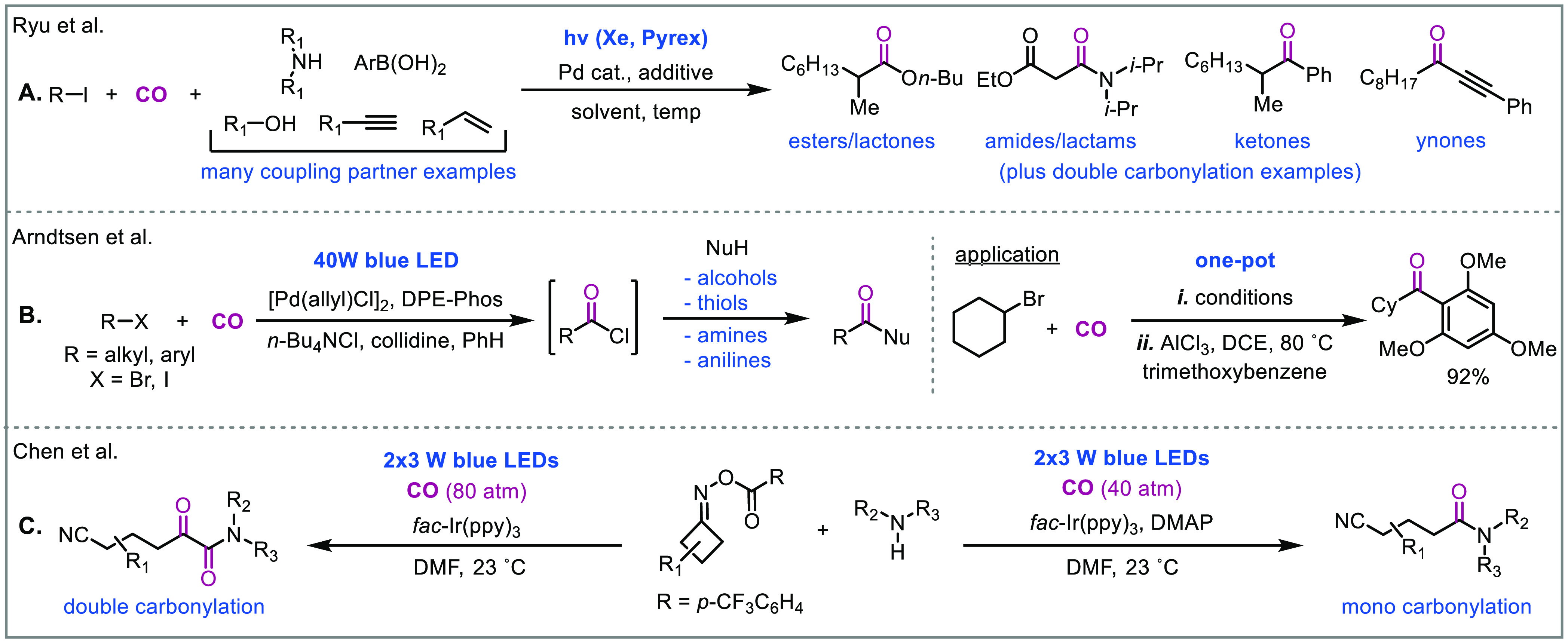
Examples of photocatalytic carbonylative transformations.

Numerous palladium-catalyzed carbonylation methods have been developed for activated halides (i.e., aryl, benzyl, allyl, vinyl halides). On the other hand, alkyl halides are notoriously challenging carbonylation substrates due to their slow rates of oxidative addition and susceptibility to β-hydride elimination which can isomerize the alkyl metal species.^42^ Unfortunately, catalyst optimization is usually not straightforward for these systems since improving the oxidative addition step often hinders reductive elimination preventing catalytic turnover. To overcome this limitation, photocatalytic carbonylation has evolved as an effective solution. In this process, light irradiation facilitates the catalytic cycle by exciting intermediates involved in both the oxidative addition and reductive elimination steps and changing two-electron redox events to single electron transfers. While very early work only encompassed the metal-free atom transfer carbonylation of alkyl iodides, the photocatalytic era began in the 1980s when the Watanabe, Suzuki, and Miyaura groups independently realized that the addition of a transition-metal catalyst could further enhance the reaction.^43^ Of note, in 2014 Ryu and colleagues demonstrated that the carbonylation of alkyl halides under ultraviolet light irradiation was dramatically accelerated by the addition of a palladium catalyst (Figure 8A).^44^ Mechanistic studies indicated interplay between radical and palladium-catalyzed steps leading to an acylpalladium intermediate that could be trapped with various nucleophiles. More recently, the Arndtsen group displayed that visible light could facilitate the palladium-catalyzed carbonylation of alkyl halides by allowing the oxidative addition and reductive elimination steps to progress with low barriers (Figure 8B).^45^ Using this method, *in situ* generated acid chlorides could be converted to ketones, esters, thioesters, and amides in excellent yields. In some studies, mixtures of mono and double carbonylation products were identified; however, the selectivity was challenging to bias. Chen, Xiao, and colleagues built on these observations and developed a switchable radical carbonylation protocol enabled by photoredox catalysis (Figure 8C).^46^ Ketoamides could be generated through the double carbonylation of nitrogen radical cations with oxime ester derived cyanoalkyl radicals. On the other hand, the mono carbonylation amide products could be generated by adding DMAP which competitively traps the cyanoalkyl acyl radical to form an electrophilic cyanoalkyl acyl-DMAP salt that can directly react with the amine.

### C–H Carbonylation

**Figure 9:**
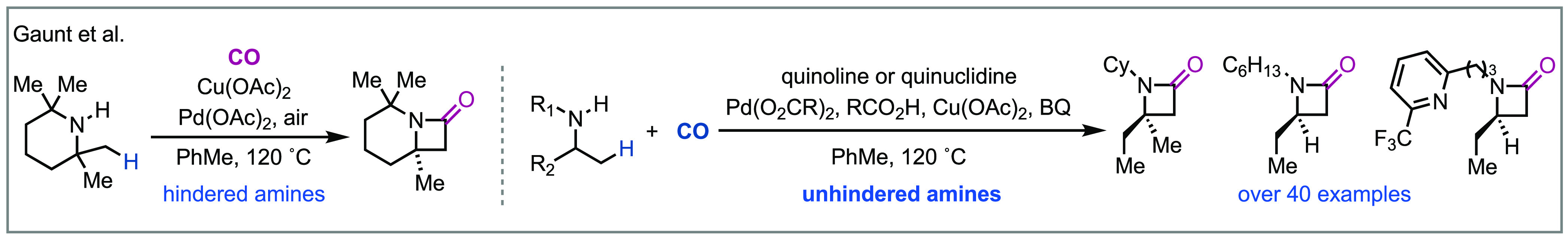
Gaunt C–H carbonylation to β-lactams.

Many electrophiles used in transition-metal-catalyzed reactions are derived from the parent C–H compound. Thus, although inherently more challenging, it is to no surprise that C–H functionalization of the parent compound has garnered much attention. Carbonylative protocols have been developed for sp, sp^2^, and sp^3^ C–H bonds, and the total synthesis community has already begun to adopt such methods.^47^ Zhao and co-workers’ Heck-type carbonylative C–H functionalization of an electron-rich arene toward the cephanolide natural products (Figure 5) is a notable example.^10^ While earlier methods mainly focused on alkynes and electron-rich arenes, more recently the C–H carbonylation of unactivated sp^2^ and sp^3^ C–H bonds has become more prominent through directing group strategies. As these methods evolve to milder conditions, broader substrate scopes and more efficient directing group strategies, they will be a powerful tool toward constructing complex natural products. In this regard, systems utilizing weakly coordinating directing groups (i.e., carbonyl, hydroxyl, and amino) have already been developed to overcome the need to install/remove strong directing groups such as pyridine-2-yl.^34^ For example, in 2014 Gaunt and colleagues reported an elegant palladium-catalyzed carbonylative C–H activation of aliphatic amines to deliver β-lactams.^48^ Notably, in addition to using weakly coordinating amines, the catalytic cycle proceeds through a four-membered ring cyclopalladation complex in contrast to the more commonly encountered five- or six-membered metallocycle intermediates. However, only hindered amines gave satisfactory yields for the transformation. To overcome this limitation, they further optimized the catalytic system for unhindered amines and showcased its potential through the synthesis of β-lactams from numerous substrate scaffolds (Figure 9).^49^ Additionally, hydrogen atom transfer (HAT) has evolved as a directing group free approach for activating C(sp^3^)–H bonds. Although less common for carbonylative processes, examples for the activation of acetonitrile, unactivated alkanes, and benzylic substrates have been reported.^34^

### Carbonylation in Flow

**Figure 10:**
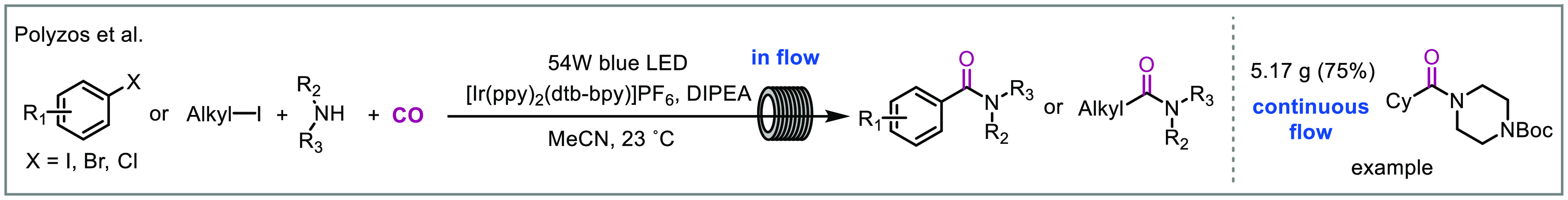
Recent example of photochemical carbonylation in flow.

In carbonylation reactions, one major limitation is that high pressures often need to be used to compensate for the low solubility of carbon monoxide. While carbon monoxide surrogates have helped overcome this limitation in some instances, using a large excess of the surrogate may be required to generate a high CO concentration which is not compatible with many systems. A greater surface area between the carbon monoxide and reaction mixture can help facilitate mass transfer at the gas–liquid interface. However, batch reactions in traditional organic chemistry glassware have very low interface areas, and utilizing volatile solvents in such systems at atmospheric pressure can lead to evaporation. Additionally, the vortex surface area formed from stirring is dependent on the exact reaction setup which adds yet another variable. To overcome this constraint, advancements in continuous flow technology have led to the development of carbonylation flow systems.^50^ In these setups, carbon monoxide delivery can be controlled by injecting controlled volumes and dissolution can be aided by increasing the flow pressure without any major safety concerns. Also, scaling up reactions is usually straightforward which will be paramount in transitioning potent lead compounds from the discovery phase to preclinical and clinical phases. Since Long and colleagues’ seminal report of carbonylative amidation in flow,^51^ many examples have entered the literature. For example, recently Polyzos and colleagues developed a visible-light-mediated carbonylative amidation of aryl, heteroaryl, and alkyl halides with aliphatic or aromatic amines in flow (Figure 10).^52^ Pleasingly, with only minor adjustments to the flow reactor the reaction could be scaled up and run continuously giving 5.17 g of product without erosion of yield. With flow chemistry already starting to enter the total synthesis realm, we anticipate that carbonylation in flow will be a common theme in complex molecule synthesis in the foreseeable future.

### Enantioselective Carbonylation

**Figure 11:**
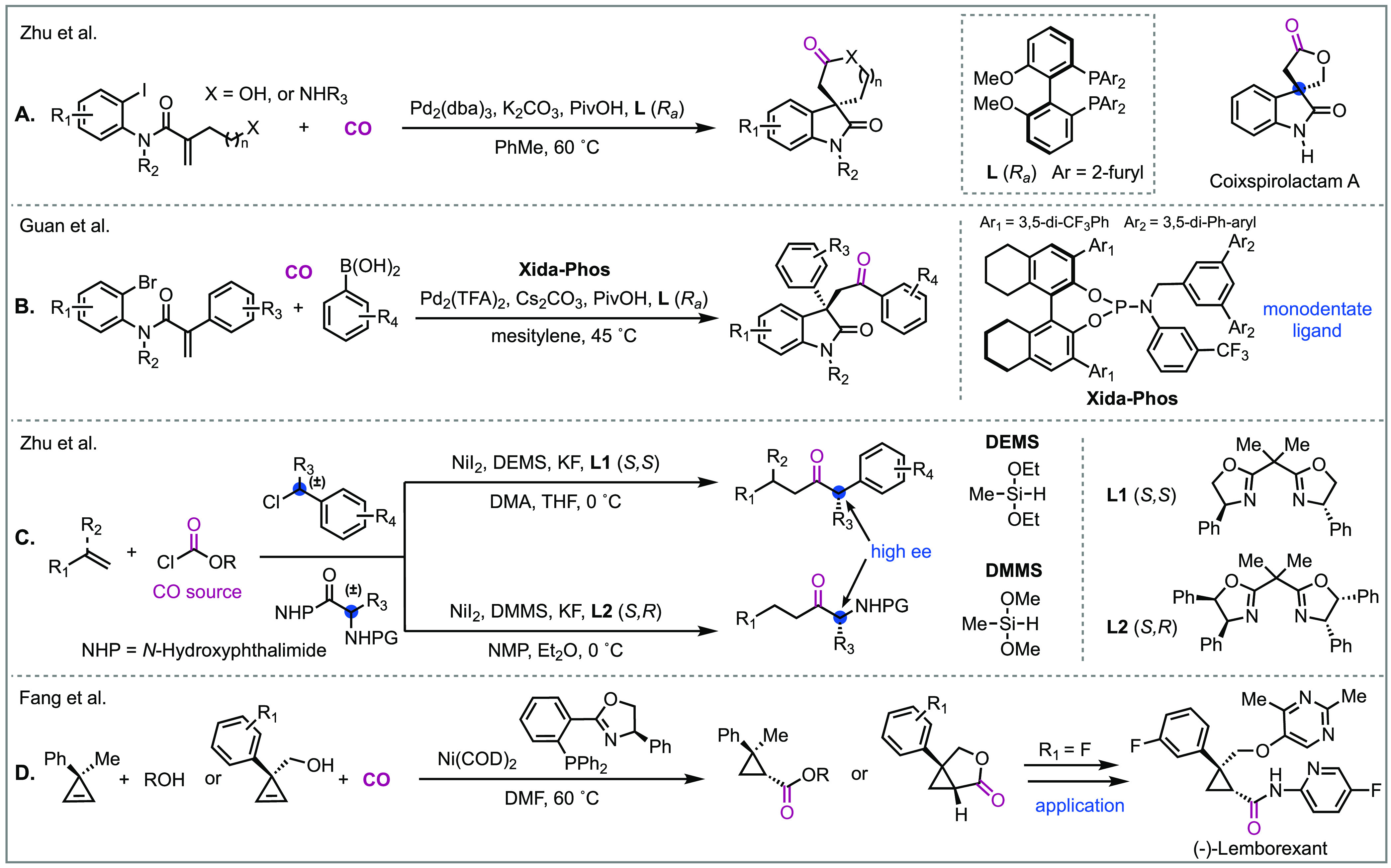
Examples of recent enantioselective carbonylations.

Nearly all complex natural products and a large portion of approved drugs contain at least one chiral center. For many of these agents, the biological potency or safety profile of one of the enantiomers is often much better, thus requiring the desired enantiomer to be isolated or enantioselectively synthesized. While enzymatic transformations and stereochemical induction via chiral pool molecules offer creative strategies, asymmetric catalysis is unparalleled in its ability to deliver a wide range of stereoselective transformations through metal and chiral ligand modifications. Although palladium-catalyzed asymmetric reactions are ubiquitous in the literature, asymmetric carbonylation variations (controlling the α-stereocenter, β-stereocenter, axial chirality, etc.) are less frequently encountered due to several challenges. Carbon monoxide is π-acidic and coordinates strongly to palladium. Thus, competitive binding of CO in place of the ligand on the palladium center can deteriorate the enantioselectivity. Additionally, the high temperatures and pressures often required for carbonylation can racemize newly formed α-stereocenters. Nevertheless, great strides have been made in palladium-catalyzed asymmetric carbonylation over the past two decades.^53^ As already showcased in this Perspective, the Heck carbonylation is a powerful method for building structural complexity and has been applied in numerous total syntheses. While the asymmetric carbonylative hydroformylation and hydroxycarbonylation of alkenes have been more broadly investigated, only very recently has progress in asymmetric Heck carbonylations been made. Notably, Zhu, Luo, and colleagues developed a palladium-catalyzed enantioselective Heck carbonylative cyclization sequence to 2-oxindole spirofused lactones and lactams from easily accessed starting materials (Figure 11A).^54^ Their conditions could deliver spirooxindole γ-and δ-lactones/lactams in excellent enantioselectivities (up to 99% ee), and they went on to use this method to synthesize a CRTH2 receptor antagonist and the natural product coixspirolactam A. Building on this work, the Guan group disclosed a similar domino enantioselective Heck carbonylation to oxindoles using a monodentate phosphoramidite ligand (Figure 11B).^55^ Instead of intramolecularly trapping the acylpalladium intermediate generated from carbon monoxide, this intermediate was reacted intermolecularly with various nucleophiles such as arylboronic acids, anilines, and alcohols to give β-carbonyl-substituted all-carbon quaternary stereocenters in high enantioselectivity. To showcase the method, they completed an asymmetric synthesis of hexahydropyrroloindole and its dimeric alkaloids. Asymmetric carbonylations with first-row transition metals are also being developed and offer a more sustainable approach to chiral scaffolds. For example, Zhu and colleagues developed a nickel-catalyzed multicomponent synthesis of α-chiral ketones by the reductive hydrocarbonylation of racemic starting materials.^37c^ Starting from racemic benzyl chlorides or *N*-hydroxyphthalimide esters, enantioenriched chiral α-aryl ketones and α-amino ketones could be delivered in excellent yields (Figure 11C). Soon after, in 2022 the Fang group developed a nickel-catalyzed asymmetric hydroaryloxy- and hydroalkoxycarbonylation of cyclopropenes (Figure 11D).^37d^ Their conditions can deliver polysubstituted cyclopropanecarboxylic acid derivatives in excellent diastereo- and enantioselectivities, and the method’s synthetic utility was demonstrated through the synthesis of the insomnia drug (−)-Lemborexant.

### C11 Incorporation via Carbonylation Reactions

**Figure 12:**
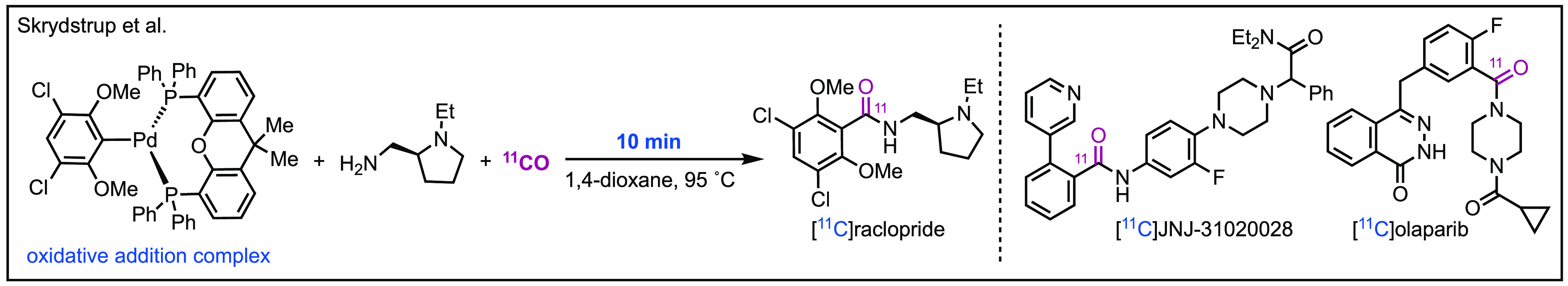
Notable [^11^C] incorporation via palladium-mediated carbonylation.

Positron emission tomography (PET) is a powerful imaging tool that is routinely used for disease diagnosis and to monitor the biodistribution of biologically active molecules. For each application, a PET tracer must be designed which is usually a small molecule labeled with a positron-emitting radioisotope. Currently, ^18^F is the most widely incorporated radionuclide, with the notable example ^18^F-Neuraceq being FDA approved for the estimation of β-amyloid neuritic density in Alzheimer’s patients. However, for the development of future tracers, incorporating an isotope based on an element that is ubiquitous among bioactive molecules would further improve the scope of tracers capable of being implemented in research and therapeutic settings. Accordingly, nearly all bioactive molecules contain carbon which has made ^11^C an attractive radionuclide in PET tracer design.^56^ While ^11^C incorporation is prevalent in the literature, most examples utilize electrophilic ^11^C methylating agents which can be incorporated through substitution reactions. Thus, this methylation technique relies on the bioactive molecule having a methylated heteroatom. Developing new synthetic incorporation methods that do not rely on methylation will be monumental in expanding the scope of ^11^C tracers. Carbonylation with ^11^CO offers a novel incorporation strategy to carbonyl containing bioactive molecules. However, due to the short half-life of ^11^C (20 min) carbonylation reaction times must be significantly short, which restricts standard carbonylation protocols from being implemented. Significantly, in 2014 Skrydstrup and colleagues utilized palladium-aryl oxidative addition complexes as stoichiometric reagents for ^11^CO carbonylation.^57^ Using this approach, several therapeutically relevant [^11^C]-pharmaceutical agents could be produced in synthetically useful yields and purity including [^11^C]raclopride, [^11^C]olaparib, and [^11^C]JNJ-31020028 (Figure 12).

### Carbonylation for Building Structural Complexity

**Figure 13:**
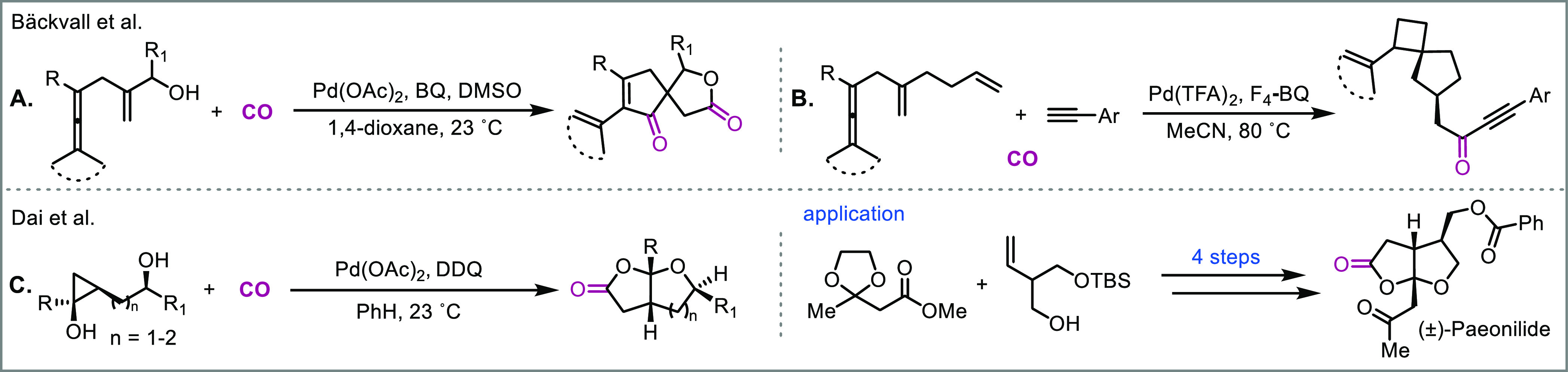
Building structural complexity through palladium-catalyzed carbonylations.

Natural products are a rich source of bioactive molecules and offer advanced starting points for therapeutic compound identification. Accordingly, many approved drug molecules are natural products or derived from a natural product scaffold. However, structural complexity is a limitation that has prevented the preparation of adequate quantities of therapeutically promising natural products for biological testing. In this Perspective, we have already demonstrated how palladium-catalyzed carbonylation reactions are helping overcome this barrier in complex natural product total synthesis. In several of the syntheses outlined, the carbonylation step helped accumulate gram-scale quantities of late-stage intermediates offering the potential for derivatization and further medicinal chemistry studies. The development of new carbonylation methodologies that build structural complexity rapidly will facilitate the future total synthesis of challenging natural products. In 2017, the Bäckvall group reported a palladium-catalyzed oxidative cascade carbonylative spirolactonization of enallenols (Figure 13a).^58a^ In this highly selective cascade, three new C–C bonds and one C–O bond are formed through the insertion of two CO molecules. Similarly, they also developed an intriguing carbonylation cascade of dienallenes to ynone tethered spirocyclobutenes (Figure 13b).^58b^ More recently, the Dai group reported a novel palladium-catalyzed ring-opening carbonylative lactonization of hydroxycyclopropanols to give substituted tetrahydrofuran and tetrahydropyran fused bicyclic γ-lactones (Figure 13c).^59^ They went on to use this method to achieve a concise synthesis of paeonilide.

## Conclusion

Transition-metal-catalyzed carbonylation is a powerful tool for constructing challenging chemical scaffolds. In this Perspective, we showcased how palladium-catalyzed carbonylation is continuing to transform the total synthesis landscape. In all the syntheses outlined, the carbonylation step helped rapidly build key bonds that otherwise would have taken multiple synthetic steps. Accordingly, the development of new carbonylation methodologies and technologies that build structural complexity rapidly will be monumental in facilitating the future total synthesis of complex products. For example, carbonylative C–H activation, photolytic carbonylation, and enantioselective carbonylation are starting to offer novel routes to distinct scaffolds that are difficult to access through other methods. For industry applications, many palladium-based carbonylation methods are oftentimes not feasible. To overcome this, more sustainable palladium-based methods that utilize lower catalyst loadings, benign oxidants (i.e., electrochemistry), and cheaper first-row transition-metal-based catalytic systems are being implemented. Additionally, carbonylative flow systems are being designed that will enable these methods to be utilized on industry scales. Undoubtedly, new developments in carbonylation chemistry will continue to positively impact natural product total synthesis, drug discovery and synthesis, PET imaging, material chemistry, agrosciences, and others.

## Data Availability

The data underlying this study are available in the published article.
